# A method for human teratogen detection by geometrically confined cell differentiation and migration

**DOI:** 10.1038/srep10038

**Published:** 2015-05-12

**Authors:** Jiangwa Xing, Yi-Chin Toh, Shuoyu Xu, Hanry Yu

**Affiliations:** 1 Institute of Biotechnology and Nanotechnology, A*STAR, The Nanos, #04-01, 31 Biopolis Way, Singapore 138669, Singapore; 2 Mechanobiology Institute, National University of Singapore, T-Lab, #05-01, 5A Engineering Drive 1, Singapore 117411, Singapore; 3 Department of Biomedical Engineering, National University of Singapore, 9 Engineering Drive 1 EA #03-12, Singapore 117575; 4 Singapore-MIT Alliance for Research and Technology, 1 CREATE Way, #10-01 CREATE Tower, Singapore 138602, Singapore; 5 Department of Physiology, Yong Loo Lin School of Medicine, MD9-04-11, 2 Medical Drive, Singapore 117597, Singapore; 6 Department of Biological Engineering, Massachusetts Institute of Technology, Cambridge, MA 02139, USA

## Abstract

Unintended exposure to teratogenic compounds can lead to various birth defects; however current animal-based testing is limited by time, cost and high inter-species variability. Here, we developed a human-relevant *in vitro* model, which recapitulated two cellular events characteristic of embryogenesis, to identify potentially teratogenic compounds. We spatially directed mesoendoderm differentiation, epithelial-mesenchymal transition and the ensuing cell migration in micropatterned human pluripotent stem cell (hPSC) colonies to collectively form an annular mesoendoderm pattern. Teratogens could disrupt the two cellular processes to alter the morphology of the mesoendoderm pattern. Image processing and statistical algorithms were developed to quantify and classify the compounds’ teratogenic potential. We not only could measure dose-dependent effects but also correctly classify species-specific drug (Thalidomide) and false negative drug (D-penicillamine) in the conventional mouse embryonic stem cell test. This model offers a scalable screening platform to mitigate the risks of teratogen exposures in human.

Teratogens are drugs or chemicals that can interfere with normal embryonic development and induce abnormalities in growth and functions^1^, resulting in various birth defects. Due to the complexity of embryonic developmental processes, the identification of teratogens rely mostly on animal models^2^. However, the need to reduce the time and cost associated with animal testing as well as circumvent high inter-species variability (~40%) in teratogenic response^3^ have galvanized the development of alternative *in vitro* models, especially those based on human pluripotent stem cells (hPSCs). The hPSC-based testing models developed so far employed temporally-controlled differentiating stem cell cultures using either directed differentiation (*i.e.,* differentiation into mesoendodermal^4^, neural^5^ or cardiac cells^6^) or random differentiation in embryoid bodies^7^. Measurements of molecular biomarkers by gene expression^4^,^5^,^7^, flow cytometry^6^, or metabolite detection^8^,^9^ were used to determine the teratogenic potential of a compound.

While measuring the temporal expression of molecular biomarkers, such as transcription factors, surface markers or secretory proteins, are fairly successful in predicting drug-induced toxicity on terminally differentiated cells^10^,^11^, their utility in detecting teratogenic effects of compounds has been limited partially due to the transient, complex and spatially organized nature of molecular signaling events during embryonic development. Therefore, a small set of biomarkers cannot adequately describe developmental processes. Embryonic development is characterized by spatio-temporally regulated cell differentiation and tissue morphogenesis, which involves collective cell migration^12^,^13^. Spatio-temporally regulated differentiation and morphogenesis are important in collectively forming developmental structures, such as the primitive streak, at the desired time and place during embryonic development^13^, which are sensitive to disruption by teratogens. We hypothesize that constructing a spatial pattern of cell differentiation and migration in hPSC cultures can provide a sensitive assay for detecting the teratogenic potential of compounds *in vitro*.

Asymmetries in both mechanical and biochemical environmental cues have been shown to play important roles in the spatial patterning of cell differentiation and collective cell migration both *in vivo*^13^,^14^,^15^,^16^ and *in vitro*^17^,^18^,^19^. Here, we used the inherent mechanical asymmetry in a micropatterned hPSC (μP-hPSC) colony as a simple and robust means to spatially localize the mesoendoderm differentiation of hPSCs and allowed them to undergo collective cell migration. Cells at the periphery of the colony preferentially expressed the mesoendoderm marker, BRACHYURY (T) after one day of differentiation. These mesoendoderm cells underwent collective cell migration to eventually form a multicellular annular pattern on day 3. In the presence of known teratogens, the formation of the annular mesoendoderm pattern was disrupted in a dose-dependent manner. Quantitative analysis of the mesoendoderm morphologic features across different compound treatment groups using feature clustering and one-way analysis of variance (ANOVA) could successfully distinguish known teratogens from the non-teratogens and avoid inter-species variation when compared with the traditional mouse embryonic stem cell test (mEST).

## Results

### Formation of an annular mesoendoderm pattern by spatially directed differentiation and collective cell migration in μP-hPSC colonies

**Figure 1:**
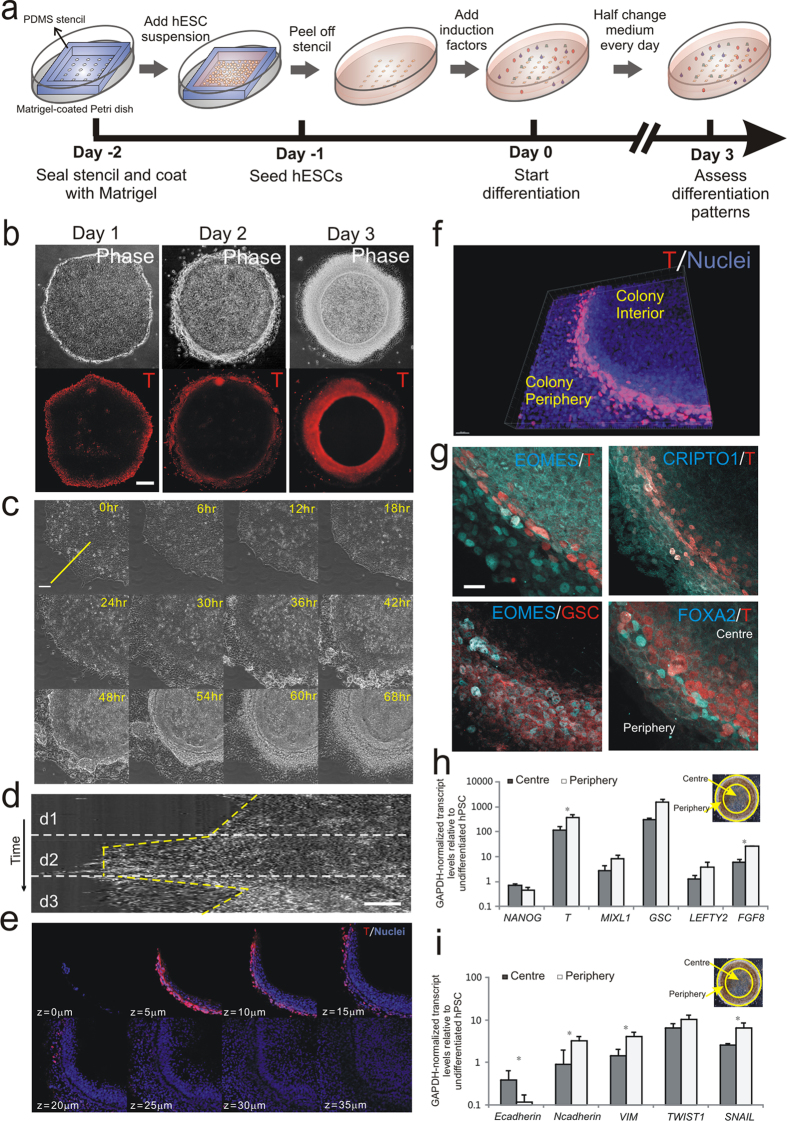
Formation of annular mesoendoderm pattern in μP-hPSC colony. (**a**) Schematic depicting micropatterning of hPSC colonies and mesoendoderm induction. (**b**) Phase and immunofluorescence images of mesoendoderm marker Brachyury (T) 1–3 days post mesoendoderm induction. Scale bar, 200 μm. (**c**) Montage from a 3-day phase imaging on a quarter section of a circular μP-hPSC colony. Scale bar, 100 μm. (**d**) Kymograph analysis showing the movement of cells along the yellow line shown in (**c**) throughout the 3-day live imaging time frame. Scale bar, 50 μm. (**e**, **f**) Confocal z-stack sections of T (red) and cell nuclei (blue)-labeled multicellular annular structure (**e**) and its 3-D reconstruction image (**f**) on day 3. Scale bar, 30 μm in (**f**). (**g**) Immunofluorescence images of mesoendoderm markers EOMES, CRIPTO1 GSC and FOXA2. Scale bar, 20 μm. (**h**,**i**) Gene expression levels of mesoendoderm markers (**h**) and EMT markers (**g**) in colony centre and periphery on day 3 relative to undifferentiated hPSCs. Data are average ± s.d. of three experiments with duplicate samples. *, p < 0.05 in paired t-test. Insets, phase image showing colony periphery and centre.

Our goal is to spatially organize cellular events (*i.e.* differentiation and cell migration) characteristic of embryonic development in hPSC cultures to assay for drug induced teratogenic effects. We leveraged on asymmetry in the mechanical environment imposed by cell micropatterning to drive differential stem cell fates^19^. We have previously shown that differential cell-matrix and cell-cell mediated adhesions between the periphery and interior regions of a hPSC colony resulted in their preferential differentiation at the colony periphery^20^. Therefore, by controlling the geometry of hPSC colony, we can prospectively determine the spatial organization of the differentiated cells. Circular micropatterned human pluripotent stem cell (μP-hPSC) colonies were generated by seeding hPSCs onto circular Matrigel islands of 1 mm in diameter that were patterned with a polydimethylsiloxane (PDMS) stencil (Fig. 1a; Supplementary Fig. S1). The surrounding substrate was passivated to constrain outgrowth of the μP-hPSC colonies. Cells in μP-hPSC colonies could maintain pluripotency and show similar gene and protein expression levels compared to conventionally cultured hPSCs cultured in mTeSR^TM^1 maintenance medium (Supplementary Fig. S2). Immunofluorescence staining showed that cells were positive for the pluripotency-associated transcription factors OCT4 and NANOG, and surface markers TRA-1–60 and SSEA-4 (Supplementary Fig. S2). Compared with unpatterned hPSCs in conventional maintenance culture, the μP-hPSCs showed similar transcript levels of both pluripotency-associated and lineage-specific genes (Supplementary Fig. S2).

To induce mesoendoderm differentiation, which is one of the earliest developmental events, the μP-hPSC colonies were cultured in a serum-free medium containing Activin A, BMP4 and FGF2 (Fig. 1a). We monitored the expression patterns of BRACHYURY (T), an early mesoendoderm marker^21^, over three days. T was initially expressed on the periphery of the colony after one day of differentiation (Fig. 1b). By day 3, the T^+^ cells were displaced inwards by approximately 200 μm from the colony edges, and formed a 3D multicellular annular pattern (Fig. 1b). When we patterned hPSCs onto Matrigel islands of different geometries but having the same colony area as the 1 mm circular pattern, we found that the shape of the T^+^ mesoendoderm patterns corresponded to the geometries of the underlying Matrigel islands and were similarly displaced inwards from the colony periphery (Supplementary Fig. S3). However, there was no significant difference in the extent of mesoendoderm differentiation among different colony geometries (Supplementary Fig. S3). When we varied the size of the colony while keeping the same circular geometry, we observed that we could still generate an annular mesoendoderm pattern although the proportion of T^+^ cells in the colony increased (Supplementary Fig. S3). Therefore, we demonstrate that stipulating the geometry of the μP-hPSC colonies could reliably control the formation of the mesoendoderm pattern.

The consistent displacement of T^+^ cells from the colony periphery towards the interior from day 1 to day 3 after mesoendoderm induction suggested that these cells underwent collective cell migration (Fig. 1b, Supplementary Fig. S3). To verify this, a 3-day live imaging under 10X objective using phase contrast was performed to track the cell movements. Kymograph analysis gave a graphical representation of spatial position changes along a line over the three days of mesoendoderm induction^22^. Results showed that after one day of induction, periphery cells became motile and migrated out from the main colony. While T^+^ periphery cells in unpatterned hPSC colonies spread out from the colony continuously (Supplementary Fig. S4), the physical constraint of the μP-hPSC colonies allowed the periphery cells to migrate outwards for only about ~150 μm. The majority of these motile cells then established contact with each other, and migrated for about 200 μm towards in the colony interior in an amoeboid manner on top of a cell layer in contact with the underlying substrate (Fig. 1c; Supplementary Video 1). The spreading and retraction of the motile cells in the μP-hPSC colonies during the 3-day time frame was manifested as a major migratory front in the kymograph, which was consistently observed in different colonies (Fig. 1d).

To study the annular mesoendoderm pattern formed by both differentiation and cell migration, a confocal z-stack analysis of the periphery section of a T-labeled μP-hPSC colony was performed to reveal its internal structure (Fig. 1e,f). The cross section of the mesoendoderm pattern resembled a ridge-like multicellular structure (Fig. 1f), where the T^+^ cells were mainly localized on top of the ridge, and a narrow furrow a few microns in width ran underneath the ridge (Fig. 1e). This ridge-like structure appeared to resemble an invagination of the epiblast sheet^23^ seen during primitive streak formation *in vivo*, which is approximately an *in vivo* equivalent of the hPSCs-derived mesoendoderm cells at the multicellular ridge.

The protein and gene expressions of various mesoendoderm markers and epithelial-mesenchymal transition (EMT) markers were examined after three days of mesoendoderm induction to confirm that the migrating cells at the colony periphery were indeed mesoendoderm cells (Fig. 1g-i). Immunofluorescence staining showed that the mesoendoderm markers EOMES, GSC, CRIPTO, as well as the early definitive endoderm marker FOXA2 all co-localized with T at the colony periphery (Fig. 1g). RT-PCR results showed that cells at the colony periphery exhibited higher transcript levels of mesoendoderm-specific genes (*T*, *MIXL1*, *GSC*, *LEFTY2* and *FGF8*) and lower transcript levels of *NANOG* compared with cells at the colony centre (Fig. 1h). During gastrulation *in vivo*, epithelial-mesenchymal transition (EMT) interrelates mesoendoderm differentiation and subsequent cell migration^24^. Here, we found that higher expression levels of EMT markers (*N-cadherin*, *VIM*, *TWIST* and *SNAIL*) and lower expression level of *E-cadherin* were also detected at the colony periphery, where the multicellular annular pattern was located, as compared to the colony interior (Fig. 1i). These results collectively indicated that the motile cells at the colony periphery were mesoendoderm cells.

To demonstrate that the annular multicellular pattern is formed as a result of mesoendoderm cell differentiation and migration, we cultured μP-hPSC colonies in a basal differentiation medium without adding any mesoendoderm induction factors. Immunostaining results of fixed samples from day 1 to 3 showed no detectable T expression (Supplementary Fig. S5). Live imaging and kymograph analysis indicated that cell migration at the colony periphery was negligible in the absence of mesoendoderm induction (Supplementary Fig. S5; Supplementary Video 2). Cells proliferated and distributed relatively evenly throughout the whole colony during the 3-day time frame with no annular pattern being formed (Supplementary Fig. S5). These data confirmed that the formation of an annular mesoendoderm pattern in the μP-hPSC colonies was specifically a result of mesoendoderm cells differentiating and migrating in a geometrically confined space.

### Sensitivity and specificity of mesoendoderm pattern formation to teratogen treatment

**Figure 2:**
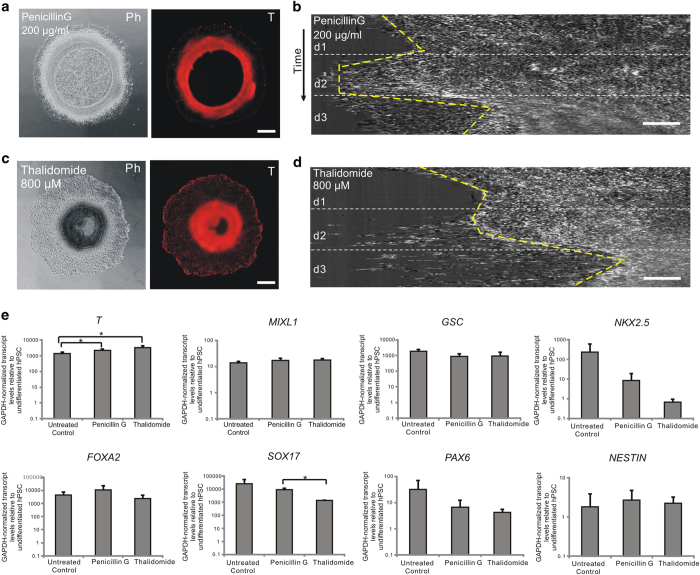
Disruption of annular mesoendoderm pattern by teratogen treatment. (**a**,**c**) Phase and T fluorescence images of μP-hPSC colonies under Penicillin G (**a**) and Thalidomide (**c**) treatment after 3-day mesoendoderm induction. Scale bar, 200 μm. (**b**,**d**) Kymographs of cell movements around colony periphery during 3-day mesoendoderm induction under Penicillin G (**b**) and Thalidomide (**d**) treatment. Scale bar, 50 μm. (**e**) RT-PCR analysis of expression levels of germ layer markers in untreated, Penicillin G-treated and Thalidomide-treated colonies on day 3 relative to undifferentiated hPSCs. Mesoendoderm markers are T, MIXL1 and GSC; mesoderm marker is NKX2.5; definitive endoderm markers are FOXA2 and SOX17; and ectoderm markers are PAX6 and NESTIN. Data are average ± s.d. of three experiments with duplicate samples. *, p < 0.05 in paired t-test.

Since the formation of the mesoendoderm pattern encompassed developmentally relevant processes (*i.e.,* differentiation and cell migration), we wanted to test if teratogens could disrupt its formation. We treated the μP-hPSC colonies with a paradigm teratogen, Thalidomide (800 μM), and a known non-teratogenic compound, Penicillin G (200 μg/ml), at their non-cytotoxic concentrations to both hPSCs and human adult fibroblasts (Supplementary Fig. S6). Although T^+^ cells could be observed in both drug-treated colonies after 3 days, the resultant mesoendoderm patterns were distinctively different (Fig. 2a,c). The colonies treated with Penicillin G had a similar annular mesoendoderm pattern to the untreated colonies, whereas the colonies treated with Thalidomide showed a much wider mesoendoderm pattern that was displaced towards the colony center (Fig. 2a,c). Live imaging and kymograph analysis showed that Thalidomide treatment could disrupt the original collective cell migration trajectory (Fig. 2d; Supplementary Video 3). While cells in Penicillin G-treated colonies underwent similar migration trajectory as that in untreated colonies, cells in Thalidomide-treated μP-hPSC colonies migrated much more towards the colony center (Fig. 2b,d; Supplementary Video 3 and 4). Since the tested concentration of both compounds was not cytotoxic to the hPSCs, the disruption of the collective cell migration process and the final morphology of the mesoendoderm pattern by Thalidomide was likely specific to its teratogenic effects. Therefore, our μP-hPSC model could successfully differentiate teratogenic compound Thalidomide from non-teratogenic compound Penicillin G.

On the contrary, it was difficult to differentiate the teratogenic effects of Thalidomide and Penicillin G by simply measuring the expression levels of molecular biomarkers for mesoendoderm differentiation. Cells from Penicillin G (200 μg/ml) and Thalidomide (800 μM) treated colonies as well as untreated control colonies were examined for the expression level of three germ layer markers using quantitative RT-PCR (Fig. 2e). There were no significant differences of the expression levels of mesoendoderm markers *MIXL1 and GSC*, mesoderm marker *NKX2.5*, endoderm marker *FOXA2,* and ectoderm markers *PAX6* and *NESTIN* between the three test groups (Fig. 2e). Both Thalidomide-treated and Penicillin G-treated colonies showed a higher expression level of mesoendoderm marker, *T,* compared to untreated control colonies. However, we could not distinguish between Thalidomide and Penicillin G-treated samples based on *T* expression levels. Thalidomide-treated samples also exhibited significantly lower expression level of definitive endoderm marker *SOX17* than Penicillin G-treated colonies. However, there was no significant difference of *SOX17* expression level for either of these two treated samples compared with untreated control colonies (Fig. 2e). Therefore, we reasoned that measuring changes in the mesoendoderm pattern, which is an assimilation of multiple cellular processes, as an assay readout for teratogenic potential, is sufficiently sensitive and may show better specificity as compared to the expression levels of a panel of molecular biomarkers for differentiation.

### A quantitative morphometric assay to classify teratogenic potential of compounds

**Figure 3:**
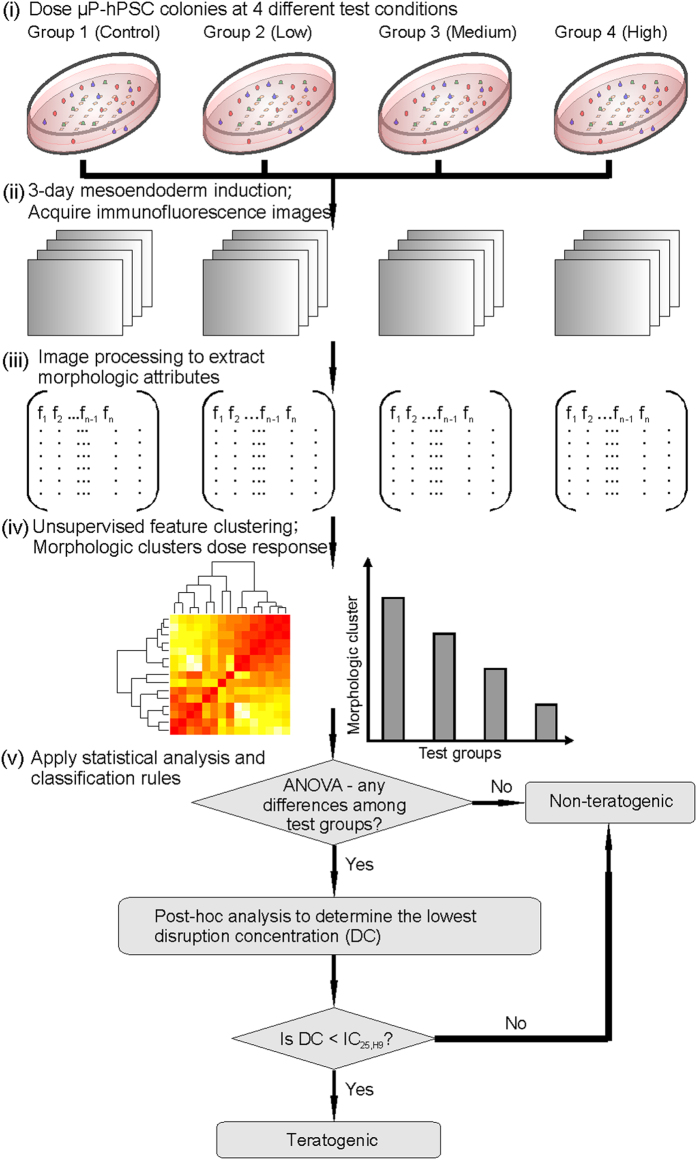
A quantitative morphometric assay for teratogen screening. Workflow for using the μP-hPSC model to determine the teratogenic potential of a test compound. Disruption concentration (DC) refers to the lowest concentration which morphologically disrupts the mesoendoderm pattern.

Since the morphological features of the annular mesoendoderm pattern in our μP-hPSC model were sensitive to teratogen treatment, we wanted to develop a quantitative assay to measure drug-induced morphological changes to the mesoendoderm pattern. The dose-dependent effect of each compound on the mesoendoderm pattern formation was determined by dosing at four test conditions: zero, low, medium and high drug concentrations (Fig. 3i). The cytotoxicity of each compound was evaluated in order to find the appropriate range of testing concentrations. Since drugs or chemicals may have different cytotoxic effects on hPSCs and human adult cells, human embryonic stem cell line, H9 and adult human dermal fibroblasts (aHDFs) were tested for cytotoxicity of each drug. The results would represent specific cytotoxicity to embryonic and adult cells respectively. Based on the cytotoxicity data, three drug concentrations (designated as low, medium and high) were selected such that the lowest concentration tested was not toxic to both H9 cells and aHDFs, and the highest concentration should not be cytotoxic to aHDFs. The tested concentration was considered as not cytotoxic if it was less than the drug’s 25% inhibitory concentration (IC_25_) to the tested cell line^25^.

A series of imaging processing and statistical analysis were developed to quantitatively measure the teratogenic effects of each drug (Fig. 3). After three days of culture in the mesoendoderm induction medium, immunofluorescence images of T were acquired (Fig. 3ii). We employed similar image quantification algorithms previously used for analyzing collagen deposition patterns in fibrotic tissues^26^ to extract 19 morphological attributes that describe the annular mesoendoderm pattern (Fig. 3iii). After excluding four extraneous attributes, which showed random trends, multivariate statistical analysis was applied to compress remaining 15 morphological attributes into indices that can indicate for drug-induced changes. Here, we used unsupervised hierarchical clustering, which is commonly used in gene microarray analysis^27^, to identify groups of morphological attributes that were affected by the drug treatment in similar ways, which we termed “morphological clusters”. A dose response plot was then generated for each morphologic cluster (Fig. 3iv). One-way analysis of variance (ANOVA) was performed to determine whether there were significant morphologic differences among test groups for each drug (Fig. 3v). If no significant differences among groups were identified in all morphologic clusters, we could directly classify the tested drug as non-teratogenic.

On the contrary, if any morphologic clusters showed significant differences among four test groups, post-hoc analysis was then performed in those morphologic clusters to confirm the ANOVA results, as well as to find the lowest concentration showing significant mesoendoderm pattern disruption compared with the non-treated control group. This concentration was defined as the disruption concentration (DC). If DC < IC_25,H9_, we can infer that the drug is teratogenic, where it affects embryonic development without being cytotoxic to embryonic cells^25^.

### Evaluation of the morphometric μP-hPSC model in classifying teratogens

We evaluated the drug testing performance of the μP-hPSC model by comparing with a well-established stem cell-based assay, the mouse embryonic stem cell test (mEST). The mEST measures whether a drug or chemical can disrupt beating cardiomyocyte formation using mouse embryonic stem cells (mESCs), and is currently one of the leading *in vitro* models being validated for teratogenicity screening^28^,^29^. Here, five drugs were selected according to the United States (US) FDA Pharmaceutical Pregnancy Risk Categories based on animal and/or human data (Supplementary Table S1). Four of the drugs are classified as teratogens *in vivo*^30^,^31^,^32^,^33^ (Category D or X) while a non-teratogenic^34^ drug *in vivo* (Category B) was included as a negative control. The mEST can only accurately classify three out of the five selected drugs (Supplementary Table 1). Thalidomide affects human but not mouse development and therefore cannot be detected in mEST^35^. D-penicillamine, on the other hand, was misclassified as non-terotogenic in mEST^36^ due to the model’s limitation in assessing only cardiogenesis endpoints^37^. By choosing these five model drugs, we aimed to evaluate whether our μP-hPSC model can potentially show better performance than the mEST.

**Figure 4:**
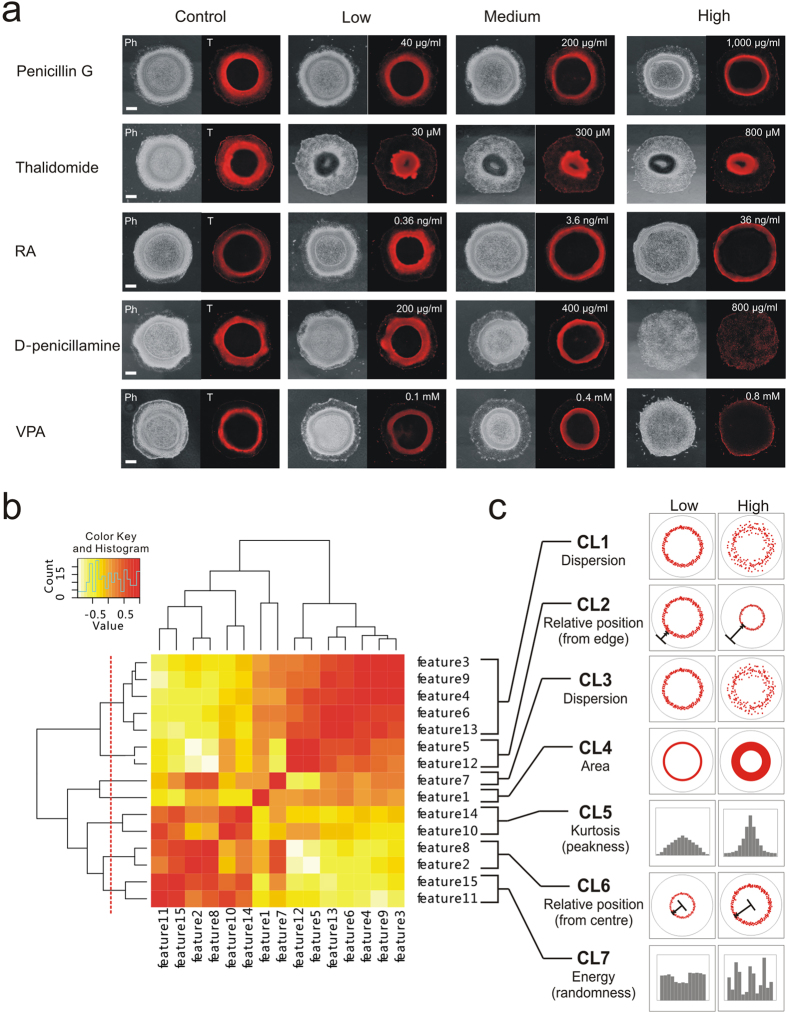
Generation of morphologic clusters by unsupervised feature clustering. (**a**) Phase and T immunofluorescence images of μP-hPSC colonies in different drug test groups on day 3. Scale bar, 200 μm. (**b**) Hierarchical clustering of morphologic attributes based on feature correlations. Dash line indicates that 7 clusters were acquired. (**c**) Graphical interpretations of the 7 morphologic clusters to describe changes to the annular mesoendoderm pattern.

First, the cytotoxicity data of each drug on both H9 cells and aHDFs were acquired to determine the low, medium and high concentrations for different test groups (Supplementary Fig. S6). Drug dosing, mesoendoderm induction as well as immunofluorescence images were acquired (Fig. 4a) and processed as described above. Seven morphologic clusters were generated by clustering the extracted fifteen morphologic attributes based on their correlations with each other (Fig. 4b). These seven morphologic clusters collectively describe the dispersion (CL1 and CL3), position (CL2 and CL6), area (CL4), kurtosis (CL5) and energy (CL7) of the T^+^ cell distribution within each μP-hPSC colony (Fig. 4c).

**Figure 5:**
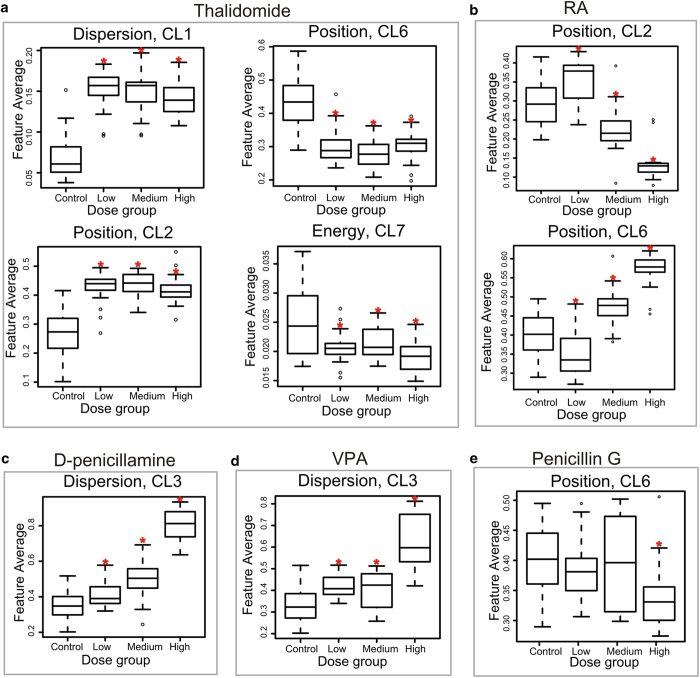
Teratogen screening results in the μP-hPSC model. (**a**-**e**) Boxplots of morphologic cluster readout, which showed clear dose-dependent disruption effects of each drug among the four test groups. The low, medium, high tested concentrations of each drug were 30 μM , 300 μM, 800 μM for Thalidomide in (**a**); 0.00036 μg/ml, 0.0036 μg/ml and 0.036 μg/ml for RA in (**b**); 200 μg/ml, 400 μg/ml and 800 μg/ml for D-penicillamine in (**c**); 0.1 mM, 0.4 mM, 0.8 mM for VPA in (**d**); 40 μg/ml, 200 μg/ml, and 1,000 μg/ml for Penicillin G in (**e**). *: p < 0.0083 in post-hoc analysis.

The readouts of each morphologic cluster across the four test groups (*i.e.* control, low, medium, high) were plotted for each drug and one-way ANOVA was performed to determine whether there were significant differences across the test groups (Supplementary Fig. S7–S11). Post-hoc analysis was performed using unpaired t-test and Bonferroni correction methods to verify the ANOVA results and determine the DC values of each drug. Our assay based on the morphologic clusters showed that Thalidomide, Retinoic acid (RA), D-penicillamine, and Valproic acid (VPA) exhibited significant dose-dependent morphologic disruptions of the mesoendoderm pattern (Supplementary Fig. S8–S11). For each drug, at least one morphologic cluster showed significant disruption to the mesoendoderm pattern in the low concentration test groups (Fig. 5a–d). Therefore, the DC values for these four drugs were 30 μM for Thalidomide, 0.36 ng/ml for RA, 200 μg/ml for D-penicillamine, and 0.1 mM for VPA (Table 1). In the case of the negative control drug Penicillin G, ANOVA results showed no significant differences among test groups in almost all morphologic clusters except CL6 (p = 0.0122) (Supplementary Fig. S7). The high concentration test group at 1,000 μg/ml showed a significant inward mesoenoderm pattern position toward the colony centre compared with zero dose control group (Post-hoc analysis, p = 0.001 < 0.0083) (Fig. 5e, Supplementary Fig. 5). Therefore, the DC for Penicillin G was 1,000 μg/ml (Table 1).

Finally we compared the DC values of each drug with their IC_25_ values to H9 cells to determine whether they are teratogenic (Table 1). For Thalidomide, RA, D-penicillamine and VPA, the DC values were all less than their corresponding IC_25,H9_ values, indicating that the disruption of the mesoendoderm pattern was likely mediated by alterations to differentiation and migration rather than cytotoxicity effects on the embryonic cells. Therefore, they were identified as teratogenic in our model. In contrast, Penicillin G had a much higher DC value compared with its IC_25,H9_ value, and was classified as non-teratogenic. Therefore, our quantitative morphometric assay based on the μP-hPSC model could correctly classify the five test compounds in accordance to their teratogenicity potential *in vivo* and showed a better performance when compared with the mEST.

## Discussion

Human-specific drug screening platforms for teratogenicity are needed to avoid inter-species variations^2^. However, current *in vitro* hPSC-based models only recapitulated temporal differentiation events^4^,^5^,^6^,^7^,^8^,^9^, and overlooked other key processes during embryonic development, including spatial organization of the differentiation and morphogenic processes. Our μP-hPSC model is the first *in vitro* human developmental toxicity screening model that recapitulated both spatially controlled differentiation and collective cell migration processes during embryogenesis. Here, we demonstrated that this model was sensitive enough to distinguish a compound’s teratogenic potential, and exhibited better selectivity to human-specific effects than the mEST.

An important consideration when assessing for teratogenic potential is the dose-dependent response^1^. The effect of a potentially teratogenic compound may not be manifested *in vivo* due to a low therapeutic dose being used^4^,^38^. Therefore, an ideal *in vitro* screening model should not only identify whether a drug is potentially teratogenic, it should also be able to detect its teratogenic effects at clinically-relevant concentrations. The drug testing results in this study demonstrated the potential of the μP-hPSC model to detect teratogenic effects of compounds at clinically relevant concentrations. In fact, when compared with the compound’s highest *in vivo* concentration (C_max_) in human plasma following therapeutic dosing, the DC values we detected for RA, D-penicillamine and VPA were already lower than or equal to their known C_max_ values, indicating their strong clinical teratogenic effects (Supplementary Table S2). In contrast, the DC value for Penicillin G was much higher than the highest clinical C_max_ value reported in literature, which was 400 μg/ml^39^, confirming that it was non-teratogenic (Supplementary Table S2).

One key feature of the μP-PSC model that will facilitate its practical application as a drug-screening platform is the consistency at which we could generate the annular mesoendoderm pattern, which provides a baseline to measure drug-induced effects. In an unpatterned hPSC colony, spatial patterns of mesoendoderm differentiation were heterogeneous and vary across cultures (Supplementary Fig. S4). The use of geometric confinement by cell micropatterning could induce reproducible spatial patterning of mesoendoderm differentiation^40^. Our μP-hPSC model not only recapitulated spatially induced mesoendoderm differentiation, but also self-organized collective cell migration within the colony, forming reproducible mesoendoderm patterns. We have shown that such annular mesoendoderm pattern could be generated with different hPSC lines (Supplementary Fig. S12). Compared with other methods of creating environmental gradient to pattern stem cell fates, such as microfluidic patterning of soluble factors^41^,^42^ or micropatterned feeder cells^43^ or hydrogels^44^, the μP-hPSC approach is straightforward to implement, readily scalable, and is more amenable to high content imaging for downstream data collection and analysis. The scalability and robustness of the μP-hPSC model will facilitate future systematic validation with a large number of test compounds, and the establishment of an animal-alternative screening platform to detect the teratogenicity potential of chemical compounds in human.

## Methods

### Cell maintenance and differentiation

All the three hPSC lines, which includes human embryonic cell lines H9 and H1, and human induced pluripotent stem cell IMR90 were obtained from WiCell Research Institute, Inc. (Madison, WI, USA) and followed the same maintenance and differentiation protocols. Cells were cultured in hPSC-qualified Matrigel^TM^ (354277, BD Biosciences) coated cell culture plates using mTeSR^TM^1 medium (05850, StemCell^TM^ Technologies). Mechanical scraping was applied during normal passaging in order to only get undifferentiated hPSC colonies after Dispase (07923, StemCell^TM^ Technologies) treatment. To induce mesoendodermal differentiation, cells were cultured in basal STEMdiff^TM^ APEL^TM^ medium (05210, StemCell^TM^ Technologies) supplemented with 100 ng/ml Activin A (338-AC-025, R&D Systems), 25 ng/ml BMP4 (314–BP–010, R&D Systems) and 10 ng/ml FGF2 (233–FB–025, R&D Systems).

The adult human dermal fibroblast (aHDF) was obtained from Lonza (Singapore) and cultured in DMEM high glucose medium (10569–010, Gibco) supplemented with 10% FBS (SV30160.03, Thermo Scientific Hyclone) and 1% Pen-Strep (09367-34, Nacalai Tesque). To get single cell suspension for cell seeding, the cells were washed with 1X PBS three times and treated with 0.25% Trypsion-EDTA (25200-114, Gibco) at 37 °C for 3–4 min.

### Fabrication of PDMS stencils for micropatterning

The polydimethylsiloxane (PDMS) stencil composed of a PDMS gasket and a thin PDMS sheet with cut micropatterns, both of which were designed with L-edit Pro software (Tanner, USA). The micropatterns used in this study include circles, squares, rectangles and semi-circular arcs with areas of 7.85 × 10^5 ^μm^2^ and 1.96 × 10^5 ^μm^2^. For teratogen screening, only circles with area of 7.85 × 10^5 ^μm^2^ were used, which corresponds to 1 mm in diameter. A laser-cutter (Epilog Helix 24 Laser System, USA) was used to cut the designed patterns on a 127 μm thick PDMS sheet (Specialty Silicone Products Inc.), then the PDMS sheet was bonded to a laser-cut, 2mm thick PDMS gasket using liquid PDMS and baked at 60 °C for 3–4 hr to finally get the PDMS stencil for micropatterning (Supplementary Fig. S1). The stencils were sterilized by autoclaving at 120 °C for 30 min every time before use.

### Generation of μP-hPSC colonies and live imaging microscopy

The autoclaved PDMS stencil was first sealed onto a 60 mm petri dish (Nunc) using 200 μl of 70% ethanol. After drying in the cell culture hood, 450 μl Matrigel^TM^ in DMEM/F12 (11330032, GIBCO) was then added into each stencil and incubated for 5 hr at 37 °C before use. To obtain single cell suspension, the hPSC culture was treated with Accutase (SCR005, Merck Millipore) for about 10 min, and the cells were resuspended in mTeSR1 medium supplemented with 10 μM Y27632 (688000, Calbiochem, Merck Millipore). The cells were seeded onto the PDMS stencils at 100% confluence density of 4444 cells/mm^2^ and allowed to attach for 1 hr. The stencil was then removed and the unpatterned substrate was passivated by backfilling with 0.5% Pluronic F-127 (P2443–1KG, Sigma-Aldrich) in DMEM/F12. After 10 min incubation, the μP-hPSC colonies were washed 3 times with DMEM/F12 and incubated at least for 3 hr in mTeSR^TM^1 medium supplemented with 10 μM Y27632 before changed to mTeSR^TM^1 medium alone. Differentiation was initiated 24 hr post seeding and lasted for 3 days before collecting the samples. For phase contrast live imaging, images were acquired every 20 min by BioStation CT (Nikon) using 10X objective. The imaging period started since the induction of differentiation and lasted for 3 days.

### Immunofluorescence staining and fluorescence microscopy

Samples were fixed for 20 min in 3.7% paraformaldehyde, and permeabilized for 15 min with 0.5% Triton X-100 in PBS. After 3 hr incubation at room temperature (RT) in blocking buffer (2% BSA and 0.1% Triton X-100 in PBS), they were incubated overnight at 4 °C with primary antibodies (5-10 μg/ml in blocking buffer). The primary antibodies used in this study were rabbit anti-Nanog (ab21624, Abcam), goat anti-Oct4 (ab27985, Abcam), mouse anti-TRA-1-60 (MAB4360, Millipore), mouse anti-SSEA4 (90231, Millipore), goat anti-Brachyury (AF2085, R&D Systems), rabbit anti-Eomes (ab23345, Abcam), rabbit anti-Cripto1 (ab19917, Abcam), rabbit anti-FoxA2 (ab40874, Abcam), and goat anti-GSC (sc-22234, Santa Cruz Biotechnology). The samples were washed 4 times with 15 min interval before adding secondary antibodies. The secondary antibodies used in this study were donkey anti-rabbit IgG-Alexa Fluor^®^ 488 (1:1000, A21206, Life Technologies), donkey anti-goat IgG-Alexa Fluor® 546 (1:1000, A11056, Life Technologies), goat anti-mouse IgM-FITC (1:500, sc-2082, Santa Cruz), goat anti-mouse IgG-FITC (1:500, sc-2010, Santa Cruz), and goat anti-rabbit IgG-Alexa Fluor® 555 (1:1000, A21429, Life Technologies). After 1 hr incubation with the secondary antibodies at RT, samples were washed for 4 times with 15 min interval and counter-stained with 10 μg/ml Hoechst 33342 (H3570, Invitrogen) for 5 min. After that, samples were washed 3 times with PBS and then mounted using Fluorsave (345789, Calbiochem, Merck Millipore). Confocal images were acquired using Zeiss LSM 5 DUO microscope (Zeiss), and immunofluorescence images of entire μP-hPSC colonies for quantitative analysis were acquired using Olympus IX81 epifluorescence microscope (Olympus, Japan) with a motorized stage (Prior Scientific).

### Drug preparation

The drugs tested in this study were Penicillin G (P3032–10MU, Sigma-Aldrich), Thalidomide (T144–100MG, Sigma-Aldrich), RA (554720-500MGCN, Merck, Millipore), D-penicillamine (P4875-5G, Sigma-Aldrich) and VPA (P4543-10G, Sigma-Aldrich). The stocks of Thalidomide and RA were dissolved in DMSO (D2650, Sigma-Aldrich), while others are dissolved in distilled water. The dilutions of Thalidomide and RA for drug treatment in our μP-hPSC model were prepared such that DMSO concentration was less than 0.25%.

### Cytotoxicity assay

The cytotoxicity test was done in 96-well tissue culture plates with 100 μl of medium with or without the drug. For each drug, 8 concentrations with 5-fold dilution were tested together with the vehicle controls. For H9 cells, the plates were coated with Matrigel^TM^ before cell seeding. 10,000 hES cells or 1,000 aHDF cells were plated into each well and cultured for 3 days in the test solution with half change of the medium with or without the drug every day. On day 3, cell viability was measured using CellTiter 96^®^ AQueous One Solution Cell Proliferation Assay (MTS, G3580, Promega). Three independent tests were done for each drug to finally acquire the cytotoxicity results. IC_25_ values were acquired from either logistic regression using OriginPro 9 or direct reading from the cytotoxicity curve.

### RNA isolation, cDNA synthesis and quantitative RT-PCR

Total RNA was extracted from the samples on day 3 of the differentiation using the RNeasy^®^ Plus Micro Kit (74034, QIAGEN) according to manufacturer’s protocol. Then cDNA was synthesized from the extracted RNA using High Capacity RNA-to-cDNA Kit (4387406, Applied Biosystems, Life Technologies). To analyze the gene expression levels of each sample, quantitative RT-PCR was performed using FastStart Universal SYBR Green Master (ROX) (04913914001, Roche) on the ABI 7500 Fast Real-Time PCR system (Applied Biosystems, Life Technologies) according to the manufacturer’s standard protocol. The relative quantitative expressions values of target genes were normalized to GAPDH and expressed relative to the levels of undifferentiated hPSCs using the ΔΔCT method. All primers used were commercially validated primers from GeneCopoeia^TM^ except for *MIXL1*, *GSC*, *E-cadherin*, *N-cadherin*, *VIM*, *TWIST* and *SNAIL*. The primer sequences/Catalogue numbers can be found in Supplementary Information (Supplementary Table S3).

### Image analysis

#### Kymograph analysis

Kymographs were generated using ImageJ (Version 1.46r, NIH) with installed MultipleKymograph plugin. After importing the time series of μP-hPSC colony images, an average intensity Z-projection image was generated. A segmented line was drawn at the region of interest (ROI) (which was the colony periphery in our study) in the Z-projection image and then restored in the original time series image window using Restore Selection Tool. After that, a kymograph could be generated with a line width of 1 using MultipleKymograph Plugin, which shows the movement of cells along the segmented line within time of interest.

#### Morphological feature extraction

Morphological feature extraction from T fluorescence images was done in MATLAB Image Processing Toolbox (Mathworks). First, the outline of the μP-hPSC colony was identified by intensity difference compared with background and its centroid position was determined. The Otsu’s method was then applied to segment the whole μP-hPSC colony into T^+^ region and T^-^ region. The relative positions/distributions of each region were acquired. All of the 19 morphological features were extracted based on the distribution of the T^+^ region, mainly including the area of the T^+^ region, relative distance of the T^+^ region to the colony centroid and outline, the standard deviation, coefficient of variance, skewness, kurtosis, entropy and energy of the distribution of the T^+^ region, etc.

#### Unsupervised Feature clustering

The feature clustering was done in R (Version 3.1.2). After excluding four extraneous features, which showed random trends, the remaining 15 features were clustered into seven morphologic clusters based on their feature correlations by hierarchical clustering using complete linkage method. Based on the clustering result, the feature average values for each morphologic cluster were calculated and plotted in boxplots.

### Statistical analysis

One-way ANOVAs were used to access the effects of test compounds on the morphologic changes of mesoendoderm patterns in R. Post-hoc analysis was performed to verify significant ANOVA results and determine DC values of each drug using unpaired t-test and Bonferroni correction methods. Since there were four test groups for each compound screening, six comparisons using unpaired t-test were generated. According to Bonferroni correction, the adjusted critical p value for 0.05 significance would be 0.05 divided by the total number of comparisons, which was 0.0083 (0.05/6). Therefore, the acquired p values by unpaired t-tests were compared with the adjusted critical p value 0.0083.

## Author Contributions

J.X., Y.C.T., and H.Y. designed the experiments. J.X.,and Y.C.T. carried out experiments and analysed the data. S.X. did the imaging processing. J.X, Y.C.T., and H.Y. wrote the manuscript.

## Additional Information

**How to cite this article**: Xing, J. *et al.* A method for human teratogen detection by geometrically confined cell differentiation and migration. *Sci. Rep.* **5**, 10038; doi: 10.1038/srep10038 (2015).

## Supplementary Material

Supplementary video 1

Supplementary video 2

Supplementary video 3

Supplementary video 4

Supplementary Information

## Figures and Tables

**Table 1:** Teratogenicity screening results in the μP-hPSC model-based quantitative morphometric assay.

**Compound**	**DC**	**IC**_**25H9**_	**Does DC < IC**_**25H9**_**?**	**In the μP-hPSC model**	**In vivo human data**	**In mEST**
Thalidomide	30 μM	>1000 μM	Yes	Teratogenic	Teratogenic^30^	N.A.
RA	0.36 ng/ml	>2000 ng/ml	Yes	Teratogenic	Teratogenic^31^	Teratogenic
D-penicillamine	200 μg/ml	278 μg/ml	Yes	Teratogenic	Teratogenic^32^	Non-teratogenic
VPA	0.1 mM	0.13 mM	Yes	Teratogenic	Teratogenic^33^	Teratogenic
Penicillin G	1000 μg/ml	787 μg/ml	No	Non-teratogenic	Non-teratogenic^34^	Non-teratogenic
