# Supplementary material for: A method for human teratogen detection by geometrically confined cell differentiation and migration
Source: Sci Rep. 2015 May 12;5:10038. doi: 10.1038/srep10038 (PMC4428054; doi:10.1038/srep10038)
Supplement: Supplementary Information [file srep10038-s5.pdf]

Supplementary Information

A method for human teratogen detection by geometrically confined cell differentiation and migration

Jiangwa Xing, Yi-Chin Toh, Shuoyu Xu, Hanry Yu

Supplementary Figures and Legends

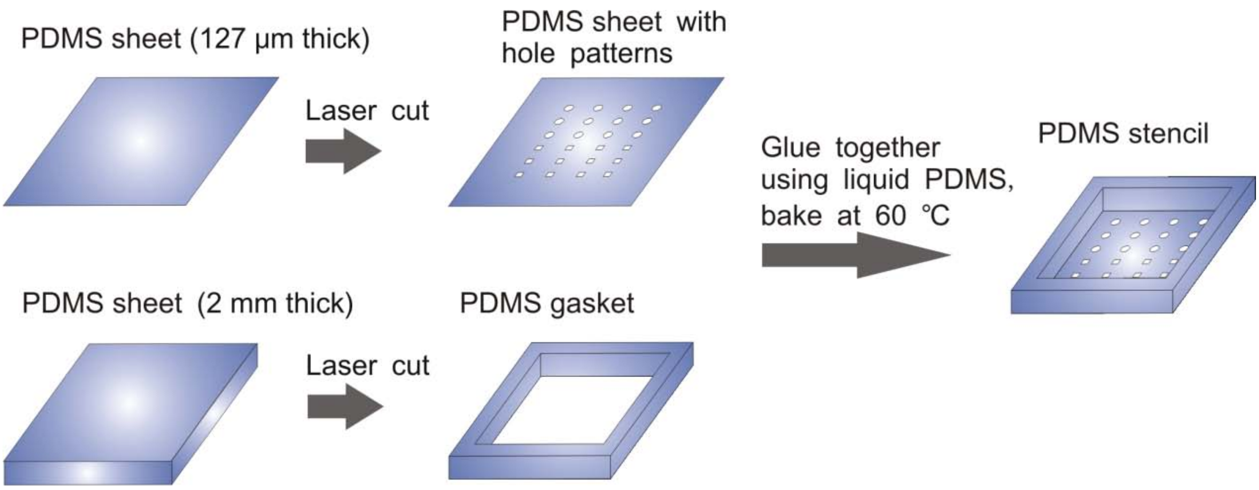

Supplementary Figure S1 | Generation of PDMS stencil for micropatterning. A laser-cutter was used to cut the designed patterns and corresponding gasket on a 127 μm thick PDMS sheet and a 2mm thick PDMS sheet respectively. After that, the two components were glued together using liquid PDMS and baked at 60°C for 3-4 hr to finally get the PDMS stencil for micropatterning.

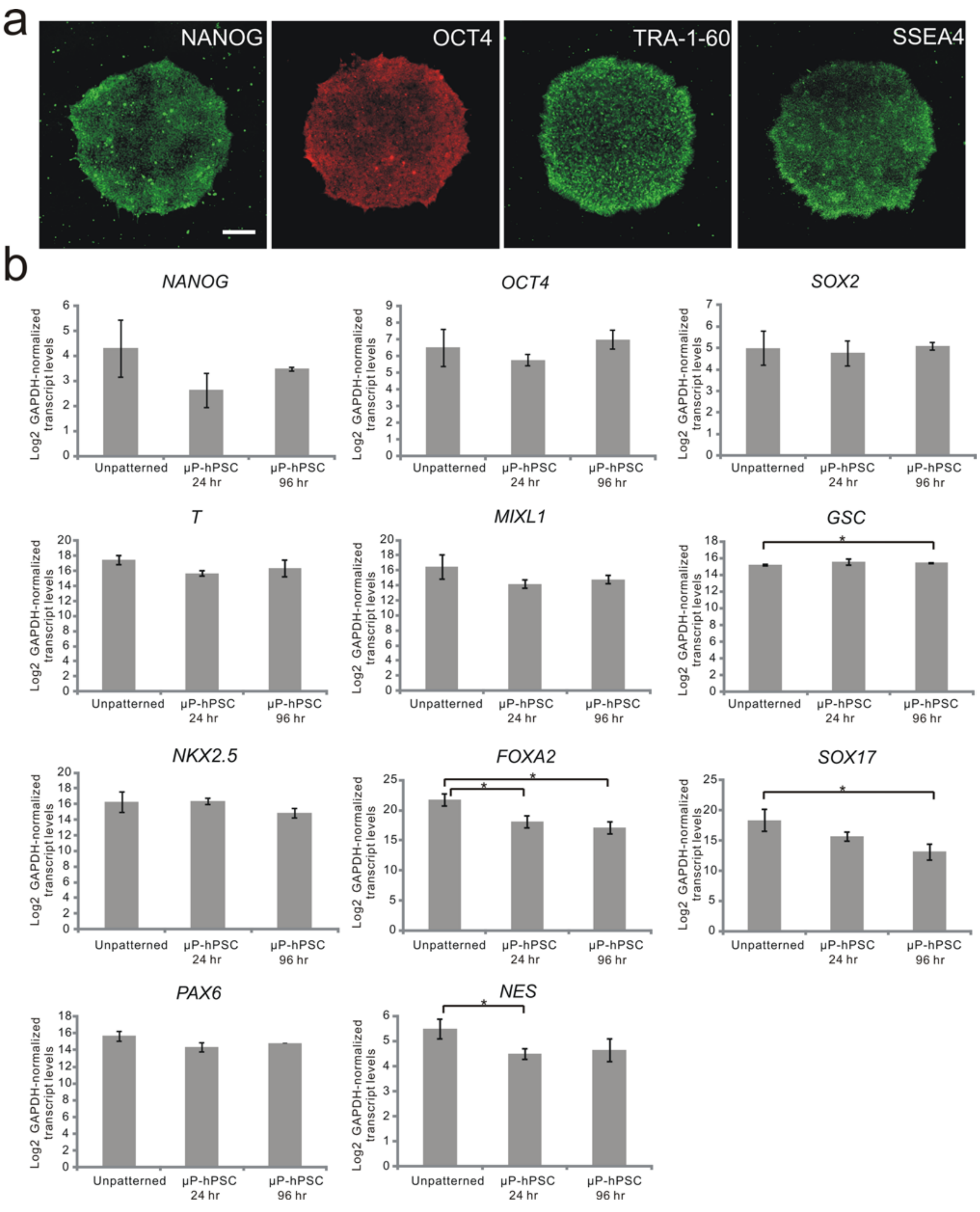

Supplementary Figure S2 | Cells in μP-hPSC colonies maintained pluripotency in mTeSR™1 maintenance medium. (a) Immunofluorescence analysis of pluripotency markers in μP-hPSC colonies 24 hr after patterning. Expression of transcription factors OCT4 and NANOG and surface antigens TRA-1-60 and SSEA4 was observed. Scale bar = 200 μm. (b) RT-PCR analysis of expression levels of pluripotency markers and lineage-specific markers in conventional unpatterned hPSCs and μP-hPSCs. Unpatterned hPSCs were lysed from normal hPSC culture when cells were

70%-80% confluent. The μP-hPSC colonies were cultured in mTeSR^{TM}1 maintenance medium and lysed for RT-PCR analysis 24 hr and 96 hr post patterning. Both unpatterned hPSCs and μP-hPSCs showed high expression levels of pluripotency markers and low expression levels of lineage-specific markers. Pluripotency markers: *NANOG*, *OCT4*, *SOX2*; Mesoendoderm markers: *T*, *MIXL1*, *GSC*, *NKX2.5*, *FOXA2* and *SOX17*: Ectoderm markers: *PAX6*, *NES* (nestin). Data are average ± s.d of three experiments with duplicate samples. *, $p<0.05$ in paired t-test.

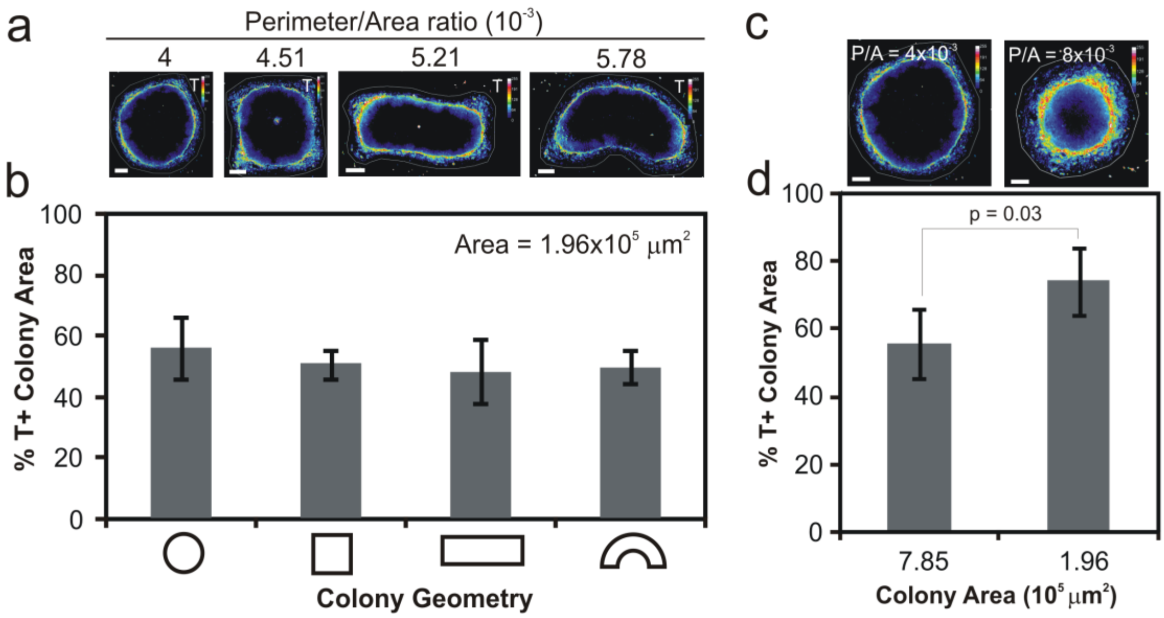

Supplementary Figure S3 | Effect of colony geometry and size on the generation of mesoendoderm patterns. (a-b) hPSCs were micropatterned on Matrigel islands of different geometries for a fixed colony area and differentiated for 3 days. (a) Intensity maps of T expression in the µP-hPSC colonies after 3 days, indicating that the shape of the mesoendoderm patterns corresponded with the geometry of the Matrigel island on which cells were patterned. (b) Quantification of T^+ colony area showing that there was no significant difference in the extent of mesoendoderm differentiation between different colony geometries (One-way ANOVA). (c-d) hPSCs were micropatterned on circular Matrigel islands of different size with different perimeter-to-area (P/A) ratios and differentiated for 3 days. (c) Intensity maps of T expression in the uP-hPSC colonies after 3 days showing similar geometries of mesoendoderm patterns. (d) Quantification of T^+ colony area showing that larger P/A ratio resulted in a significant increase in extent of mesoendoderm differentiation (Student's t-test). Data in (b,d) are average ± s.e.m of 6 colonies. Scale bars = 200 µm.

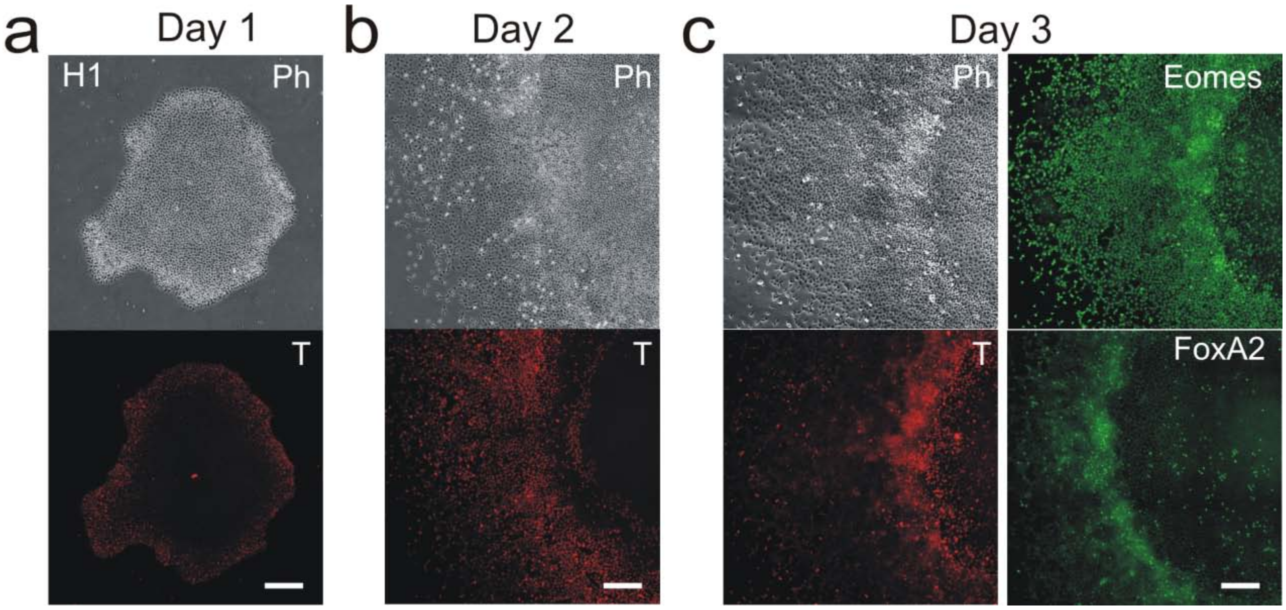

Supplementary Figure S4 | Mesoendoderm differentiation in unpatterned hPSC colonies. (a-c) Phase and fluorescent images of mesoendoderm markers for samples fixed on day 1 (a), day 2 (b) and day 3 (c). Scale bar, 200 μm.

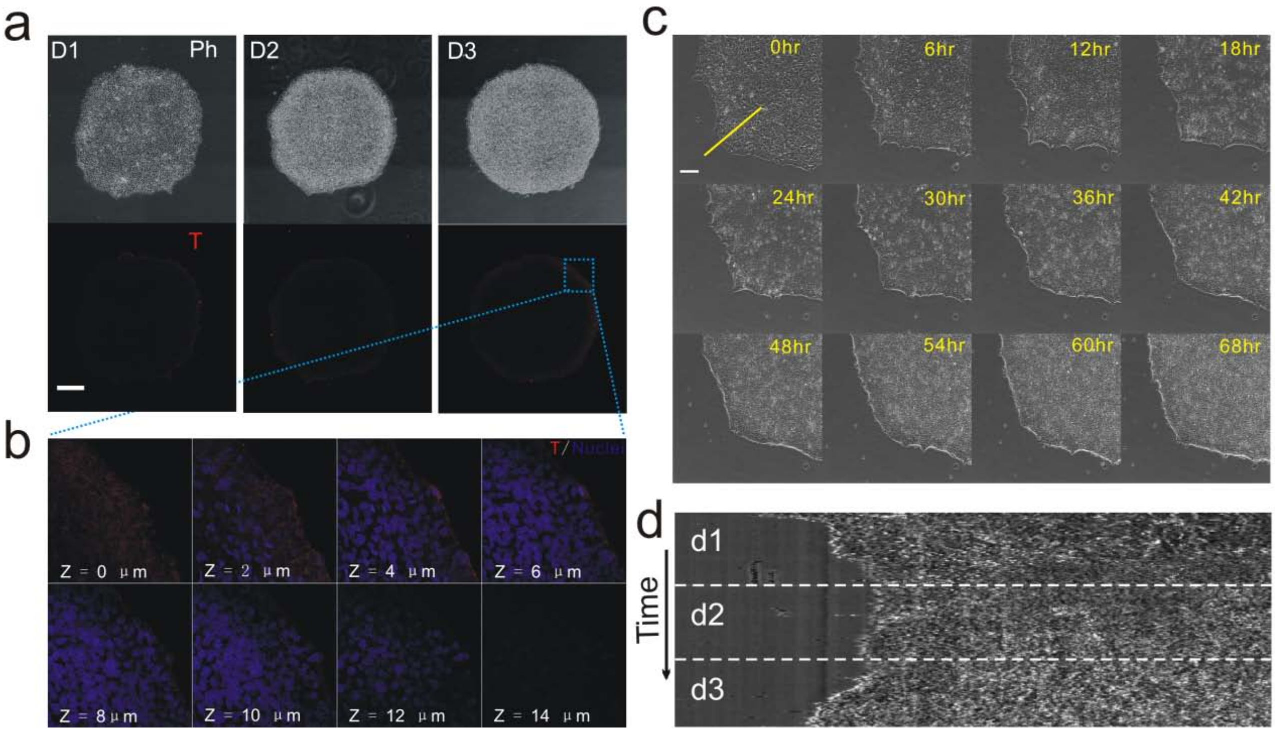

Supplementary Figure S5| No similar annular mesoendoderm pattern formed after 3-day culture in basal STEMdiff™ APEL™ medium. (a) Phase and fluorescent mesoendoderm marker T images on day 1 to day 3. Scale bar, 200 μm. (b) Confocal z-stack images of T and cell nuclei within a μP-hPSC colony fixed on day 3. (c) Montage from a 3-day phase imaging on about one quarter of a circular μP-hPSC colony. Scale bar, 100 μm. (d) Kymograph analysis showing the movement of cells along the yellow line shown in (c) throughout the 3-day live imaging time frame.

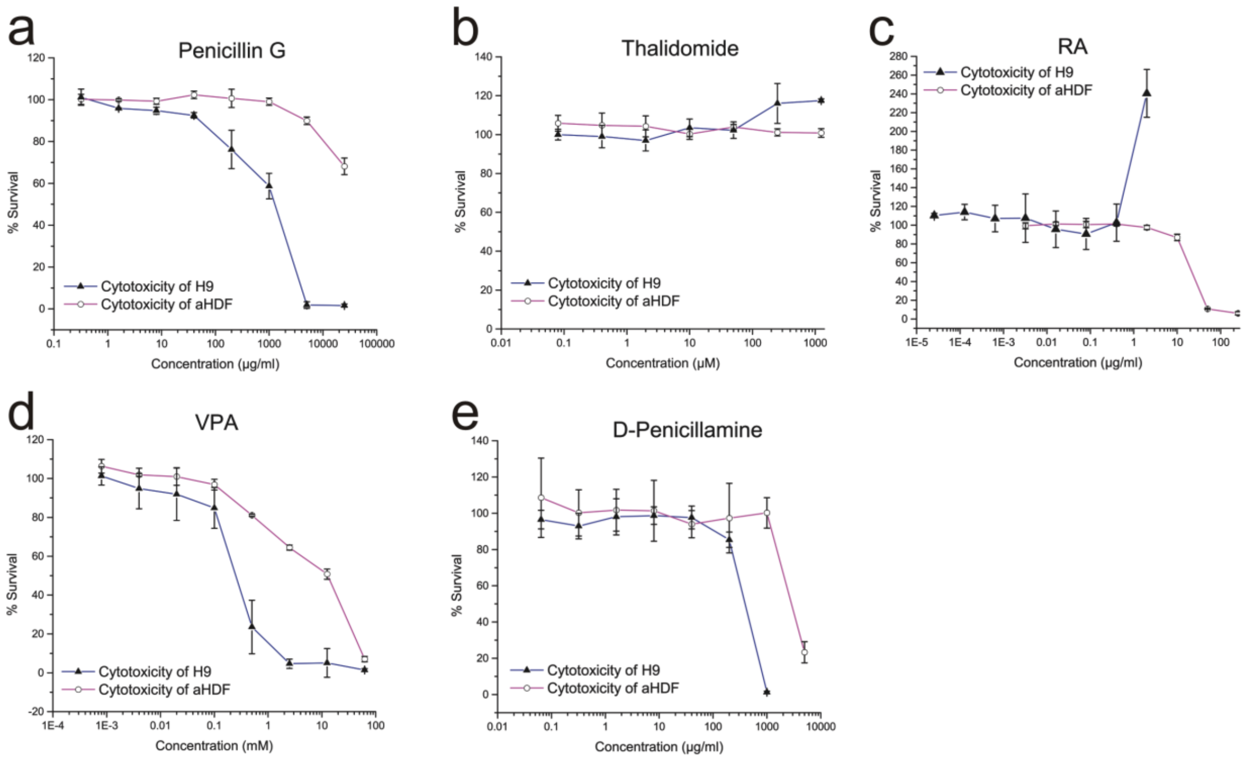

Supplementary Figure S6 | Cytotoxicity results of the five tested drugs. (a-e) Cell viability curves for drug treatment in H9 human embryonic stem cells (blue line) and adult human adult fibroblasts (aHDFs) (pink lines) (n = 3).

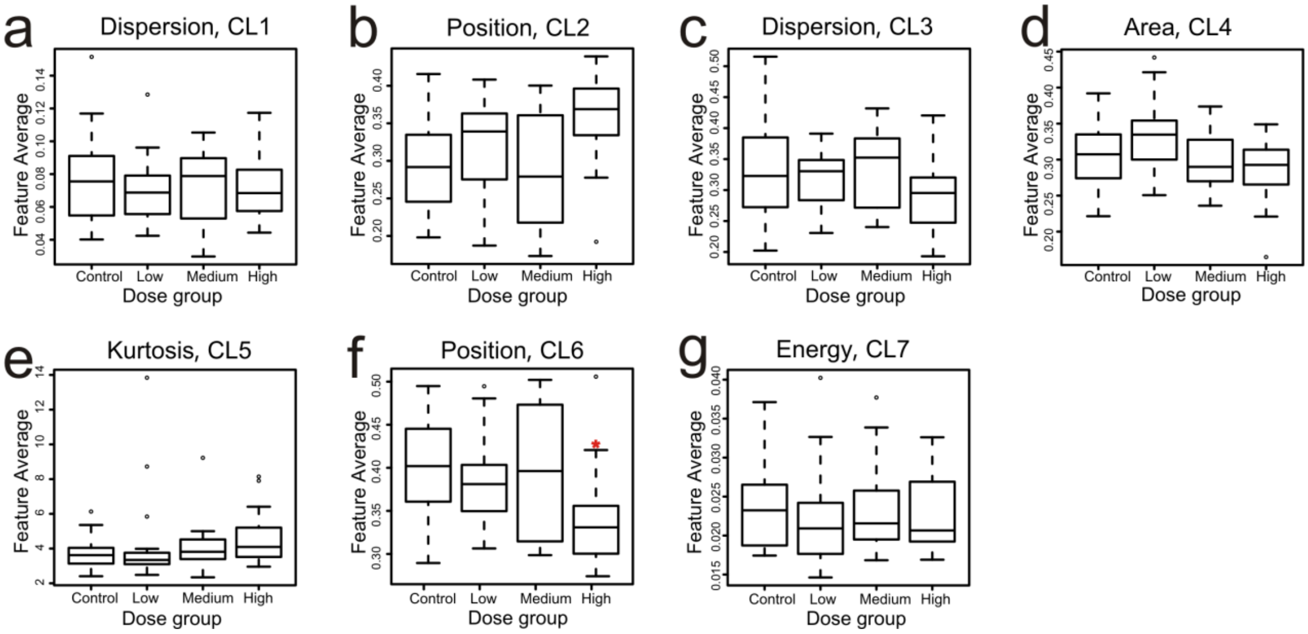

Supplementary Figure S7 | Boxplots of morphologic cluster readout in Penicillin G test groups.
*: p<0.0083 in post-hoc analysis. Low: 40 µg/ml ; Medium: 200 µg/ml; High: 1000 µg/ml

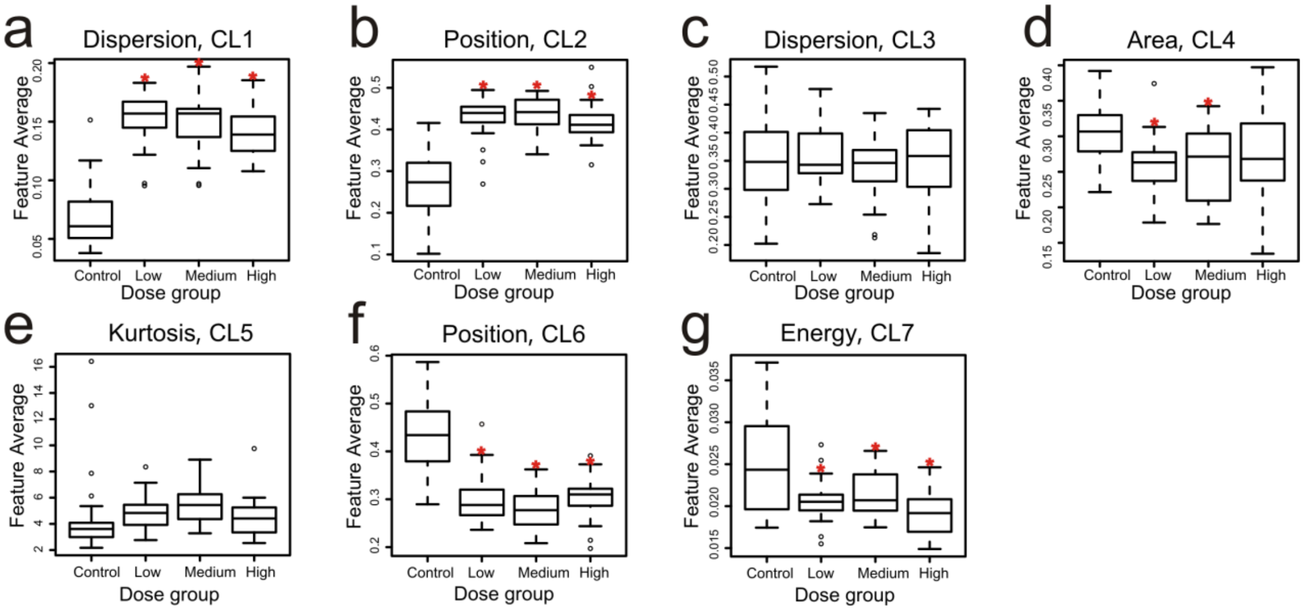

Supplementary Figure S8 | Boxplots of morphologic cluster readout in Thalidomide test groups. Low: 30 µM ; Medium: 300 µM; High: 800 µM.

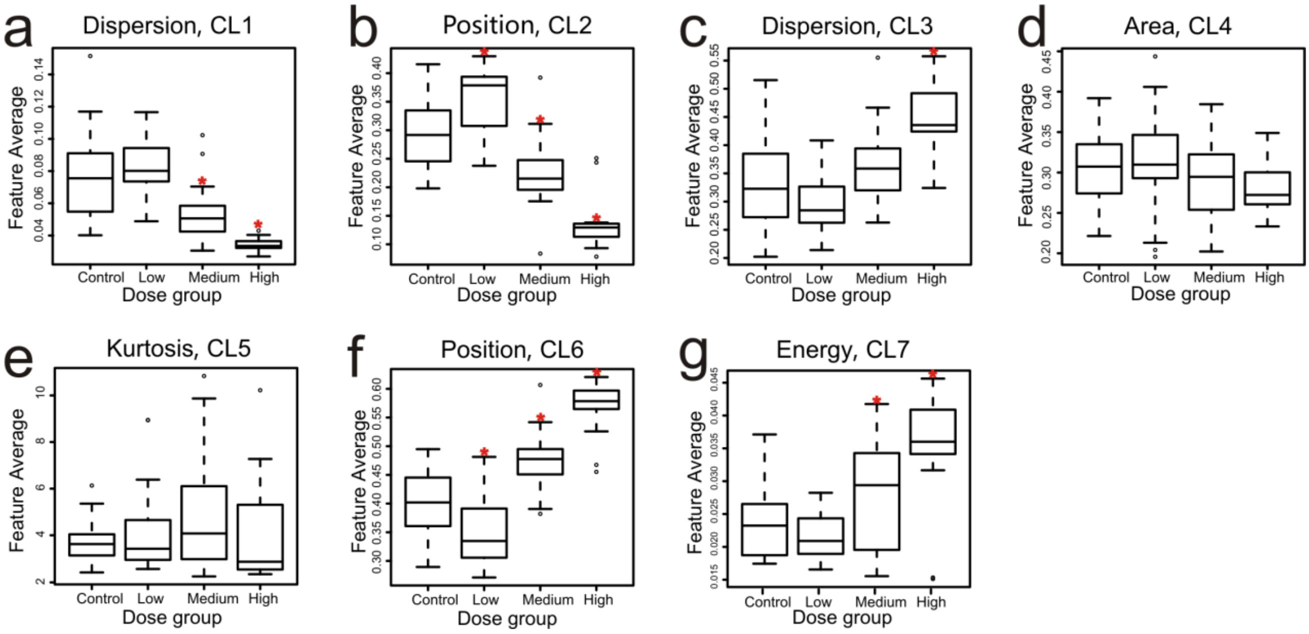

Supplementary Figure S9 | Boxplots of morphologic cluster readout in RA test groups. Low: 0.00036 µg/ml ; Medium: 0.0036 µg/ml; High: 0.036 µg/ml.

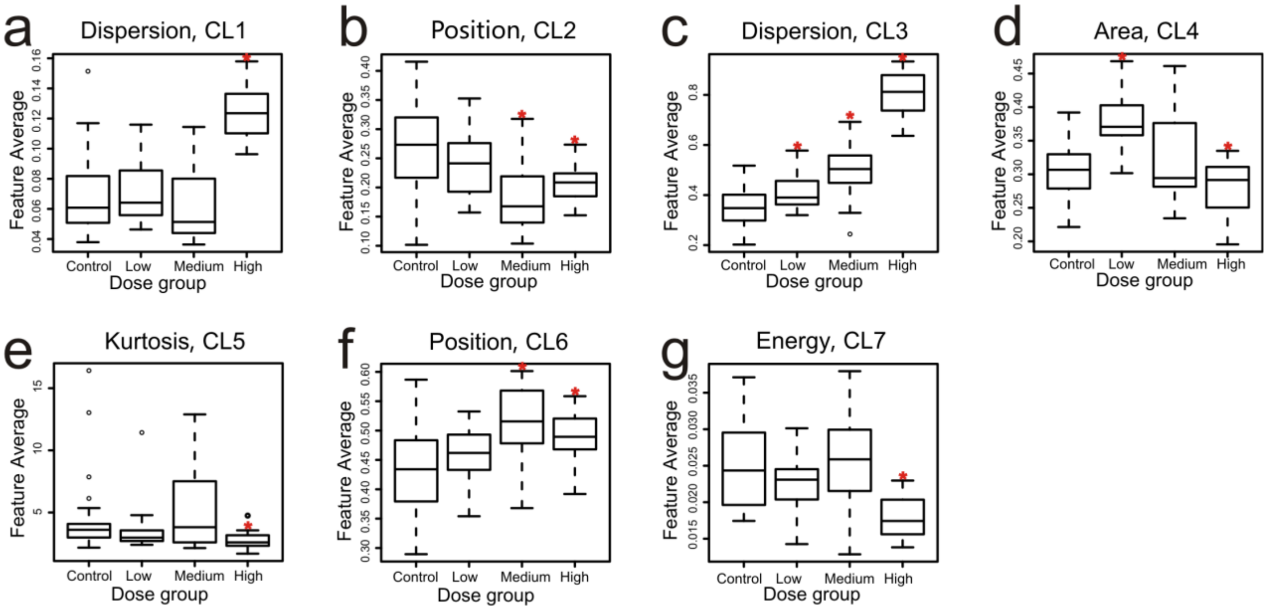

Supplementary Figure S10 | Boxplots of morphologic cluster readout in D-penicillamine test groups. Low: 200 μg/ml ; Medium: 400 μg/ml; High: 800 μg/ml.

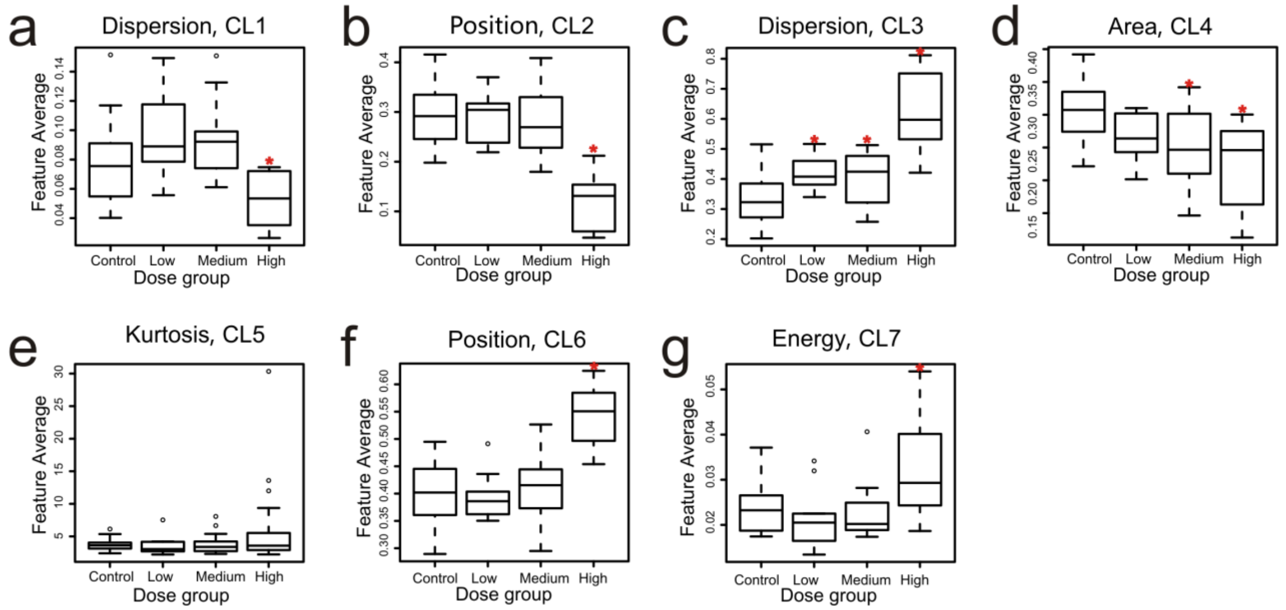

Supplementary Figure S11 | Boxplots of morphologic cluster readout in VPA test groups. Low: 0.1 mM; Medium: 0.4 mM; High: 0.8 mM.

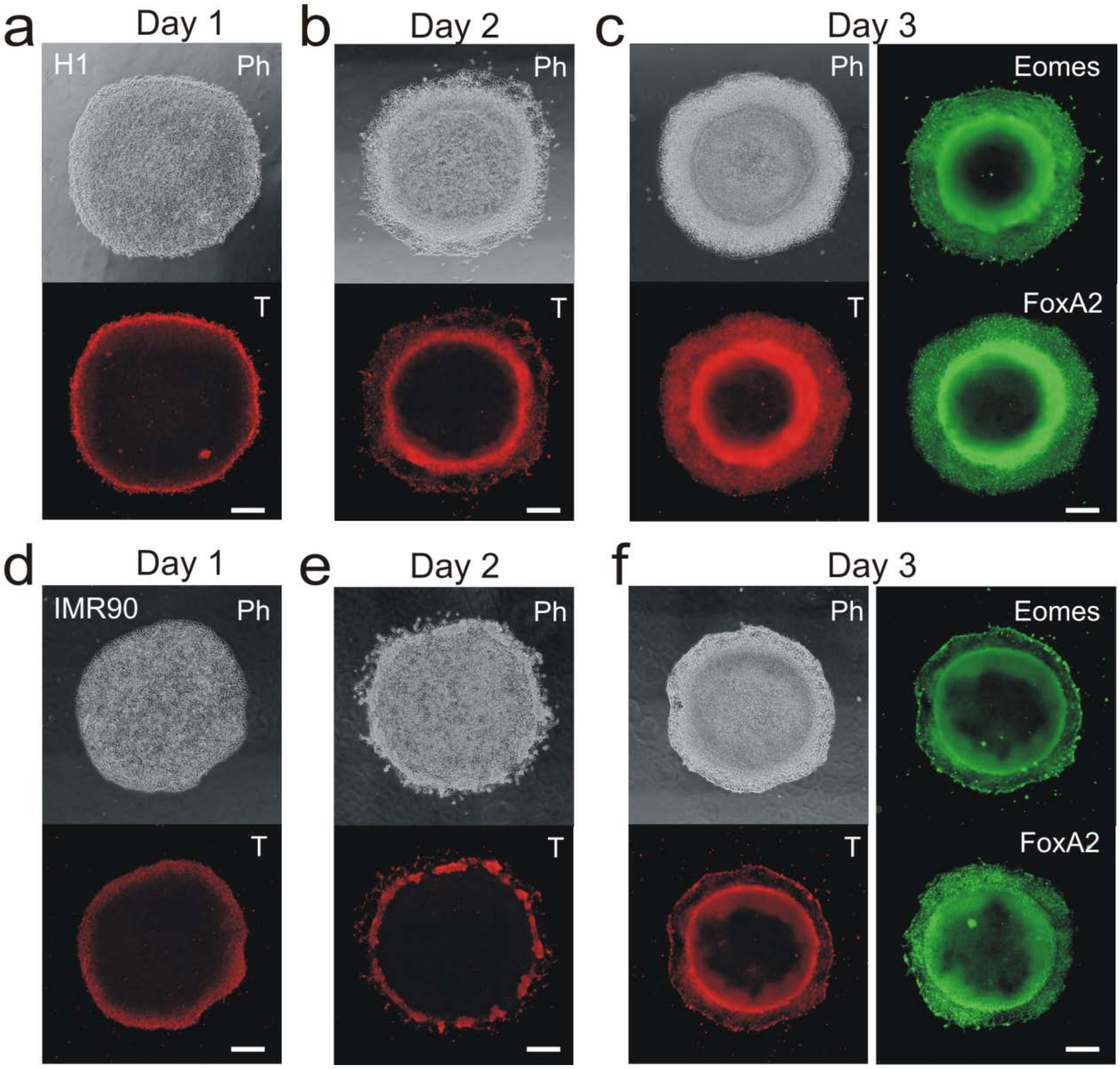

Supplementary Figure S12 | Generation of annular mesoendoderm pattern in H1 human embryonic stem cell and IMR90 induced pluripotent stem cells. (a-f) 3-day phase and fluorescent images of μP-hPSC colonies formed by H1 cells (a-c) and IMR90 cells (d-f). Scale bar, 200 μm.

Supplementary Tables

Supplementary Table 1 | Compound list for teratogen screening in the µP-hESC model

**No.**	**Compounds**	**Drug Application**	**US FDA Pharmaceutical Pregnancy Category***	***In vivo* human data**	**In mEST**
1	Thalidomide	Anti-nausea and sedative drug	Pregnancy risk category X	Teratogenic[1]	N.A.
2	Retinoic acid (RA)	Antineoplastic drug	Pregnancy risk category D	Teratogenic[2]	Teratogenic
3	D-Penicillamine	For Wilson's disease and heavy metal intoxication	Pregnancy risk category D	Teratogenic[3]	Non-teratogenic
4	Valproic acid (VPA)	Anticonvulsant and mood-stabilizing drug	Pregnancy risk category D	Teratogenic[4]	Teratogenic
5	Penicillin G	Antibotics, antibacterial drug	Pregnancy risk category B	Non-teratogenic[5]	Non-teratogenic

*: There are five categories. Category A indicates no pregnancy risk in human, Category B indicates no risk in animal studies, Category C shows positive pregnancy risk in animals, and Category D & X indicate clear risks in pregnant women.

Supplementary Table 2 | The DC and C_{max} values of test compounds

No.	Compounds	DC in µP-hPSC model	C_{max}	Reference
1	Thalidomide	30 µM	10.922 µM	Teo et al. (2004)[6]
2	RA	0.36 ng/ml	347 ng/ml	Muindi et al. (1992)[7]
3	D-penicillamine	200 µg/ml	200 µg/ml	Netter et al. (1987)[8]
4	VPA	0.1 mM	0.574 mM	Reed et al. (2006)[9]
5	Penicillin G	1000 µg/ml	400 µg/ml	Plaut et al. (1969)[10]

Supplementary Table 3 | Primer sequence/Catalogue number used for quantitative RT-PCR

**Primer**	**Sequence/Cat# (Genecopoeia)**
GAPDH	HQP006940
NANOG	HQP019390
OCT4	HQP013600
SOX2	HQP017628
BRACHYURY (T)	HQP017857
LEFTY2	HQP018049
FGF8	HQP005414
NKX2.5	HQP003440
FOXA2	HQP008906
SOX17	HQP016906
PAX6	HQP054281
NESTIN	HQP000899
MIXL1 FP	5’ – ATTTTCTCCCCTCTTCCAGGT – 3’
MIXL1 RP	5’ – GGCCTAGCCAAAGGTTGGAA – 3’
GSC FP	5’ – GGACTTGCACAGACAGACGA – 3’
GSC RP	5’ – GGTTCCTCTTTCTCGACCCC – 3’
E-cadherin FP	5’ – TGCCCAGAAAATGAAAAAGG – 3’
E-cadherin RP	5’ – GTGTATGTGGCAATGCGTTC – 3’
N-cadherin FP	5’ – ACAGTGGCCACCTACAAAGG – 3’
N-cadherin RP	5’ – CCGAGATGGGGTTGATAATG – 3’
VIM FP	5’ – GAGAACTTTGCCGTTGAAGC – 3’
VIM RP	5’ – GCTTCCTGTAGGTGGCAATC – 3’
TWIST FP	5’ – ATGTCCGCGTCCCACTAGCA – 3’
TWIST RP	5’ – ACGGGCCTGTCTCGCTTTCT – 3’
SNAIL FP	5’ – CACATCAGCCCCACAGGACT – 3’
SNAIL RP	5’ – CAGACAGGCCAGCTCAGGAA – 3’

Supplementary Video Legends

Supplementary Video 1 | Cell movement in untreated control µP-hPSC colony under mesoendoderm induction. 3-day timelapse video of about one quarter of a µP-hPSC colony under mesoendoderm induction. Images were taken every 20 min, and the play speed was 20 fps. Scale bar, 100 µm.

Supplementary Video 2 | Cell movement in µP-hPSC colony without mesoendoderm induction. 3-day timelapse video of about one quarter of a µP-hPSC colony cultured in basal STEMdiff^{TM} APEL^{TM} medium. Images were taken every 20 min, and the play speed was 20 fps. Scale bar, 100 µm.

Supplementary Video 3 | Cell movement in Thalidomide-treated µP-hPSC colony under mesoendoderm induction. 3-day timelapse video of about one quarter of a µP-hPSC colony under Thalidomide treatment. Cells moved much inwards toward colony centre compared with cells in untreated control colonies. Images were taken every 20 min, and the play speed was 20 fps. Scale bar, 100 µm.

Supplementary Video 4 | Cell movement in Penicillin G-treated µP-hPSC colony under mesoendoderm induction. 3-day timelapse video of about one quarter of a µP-hPSC colony under Penicillin G treatment. The collective cell migration process was similar as that of untreated µP-hPSC colonies. Images were taken every 20 min, and the play speed was 20 fps. Scale bar, 100 µm.

Supplementary References

1. Brent, R.L. & Holmes, L.B. Clinical and basic science lessons from the thalidomide tragedy: what have we learned about the causes of limb defects? *Teratology* **38**, 241-51 (1988).

2. Lammer, E.J. et al. Retinoic acid embryopathy. *N. Engl. J. Med.* **313**, 837-41 (1985).

3. Rosa, F.W. Teratogen update: penicillamine. *Teratology* **33**, 127-31 (1986).

4. Lammer, E.J., Sever, L.E. & Oakley, G.P., Jr. Teratogen update: valproic acid. *Teratology* **35**, 465-73 (1987).

5. Novartis, *Penicillin G Sodium injection*. (2009) Available at: http://www.medsafe.govt.nz/profs/datasheet/p/penicillinginj.pdf. (Accessed: 7th November 2014).

6. Teo, S.K. et al. Clinical pharmacokinetics of thalidomide. *Clin. Pharmacokinet.* **43**, 311-27 (2004).

7. Muindi, J.R. et al. Clinical pharmacology of oral all-trans retinoic acid in patients with acute promyelocytic leukemia. *Cancer Res.* **52**, 2138-42 (1992).

8. Netter, P., Bannwarth, B., Pere, P. & Nicolas, A. Clinical pharmacokinetics of D-penicillamine. *Clin. Pharmacokinet.* **13**, 317-33 (1987).

9. Reed, R.C., Dutta, S., Cavanaugh, J.H., Locke, C. & Granneman, G.R. Every-12-hour administration of extended-release divalproex in patients with epilepsy: impact on plasma valproic acid concentrations. *Epilepsy Behav.* **8**, 391-6 (2006).

10. Plaut, M.E., O'Connell, C.J., Pabico, R.C. & Davidson, D. Penicillin handling in normal and azotemic patients. *J. Lab. Clin. Med.* **74**, 12-8 (1969).
